# Balancing acts: lateral hypothalamic circuits coordinating feeding, anxiety, and social interactions

**DOI:** 10.1007/s11154-026-10036-7

**Published:** 2026-04-22

**Authors:** Tatiana Korotkova

**Affiliations:** 1 Institute for Systems Physiology, Faculty of Medicine, University of Cologne / University Clinic Cologne, Cologne, Germany; 2 Cologne Excellence Cluster on Aging and Aging-Associated Diseases (CECAD), University of Cologne, Cologne, Germany

**Keywords:** Innate behavior, Leptin, Orexin, Oscillations, Brain circuits, Optogenetics

## Abstract

Survival requires organisms to continuously balance competing motivational drives, including the need to acquire energy, avoid threat, and navigate complex social environments. Feeding, anxiety-related behaviors, and social interactions are therefore tightly interconnected, yet the neural mechanisms that coordinate these domains remain incompletely understood. The lateral hypothalamus (LH) is uniquely positioned to integrate internal metabolic signals, external environmental cues, and socially relevant information, and to translate this integrated state into appropriate behavioral responses. Once viewed primarily as a regulator of feeding, the LH is now recognized as a highly heterogeneous structure comprising intermingled neuronal subpopulations that influence reward seeking, stress responses, arousal, and social behavior. Emerging evidence indicates that these distinct but overlapping circuits dynamically allocate behavioral resources between energy acquisition, social interactions and overcoming anxiety, enabling flexible adaptation to changing internal and external demands. In this review, we discuss how LH circuits coordinate feeding, social behavior, and anxiety, and propose that this region functions as a central hub for balancing competing motivational states.

## Main text

All organisms face the continuous challenge of allocating behavior among competing needs. Feeding is essential for energy homeostasis, safety is important for survival, and social interactions are crucial for reproduction. Since fulfilling one need often comes at the expense of others, organisms must constantly make trade-offs. This raises the question of how the most urgent need is identified and prioritized at any given moment, and how a hierarchy of needs is established. Moreover, how do organisms suppress one need in order to satisfy another - for example, overcoming anxiety to seek food, or resisting hunger when a mating opportunity arises? Adaptive behavior therefore requires not only the execution of specific actions, but also the ability to prioritize, switch between, and sometimes resist competing motivational drives.

## From feeding center to motivational hub: multitasking of the lateral hypothalamus

Understanding how the brain performs this arbitration remains a fundamental challenge. While individual neural circuits underlying feeding, anxiety, or social behavior have been extensively characterized, far less is known about how these circuits are coordinated to enable flexible transitions between behaviors. The lateral hypothalamus is uniquely positioned to solve this problem. Receiving convergent metabolic, emotional, sensory, and social-related inputs, the lateral hypothalamus (LH) can integrate internal state with external conditions and translate this information into context-appropriate behavioral output in a flexible, state-depended way.

**Fig. 1:**
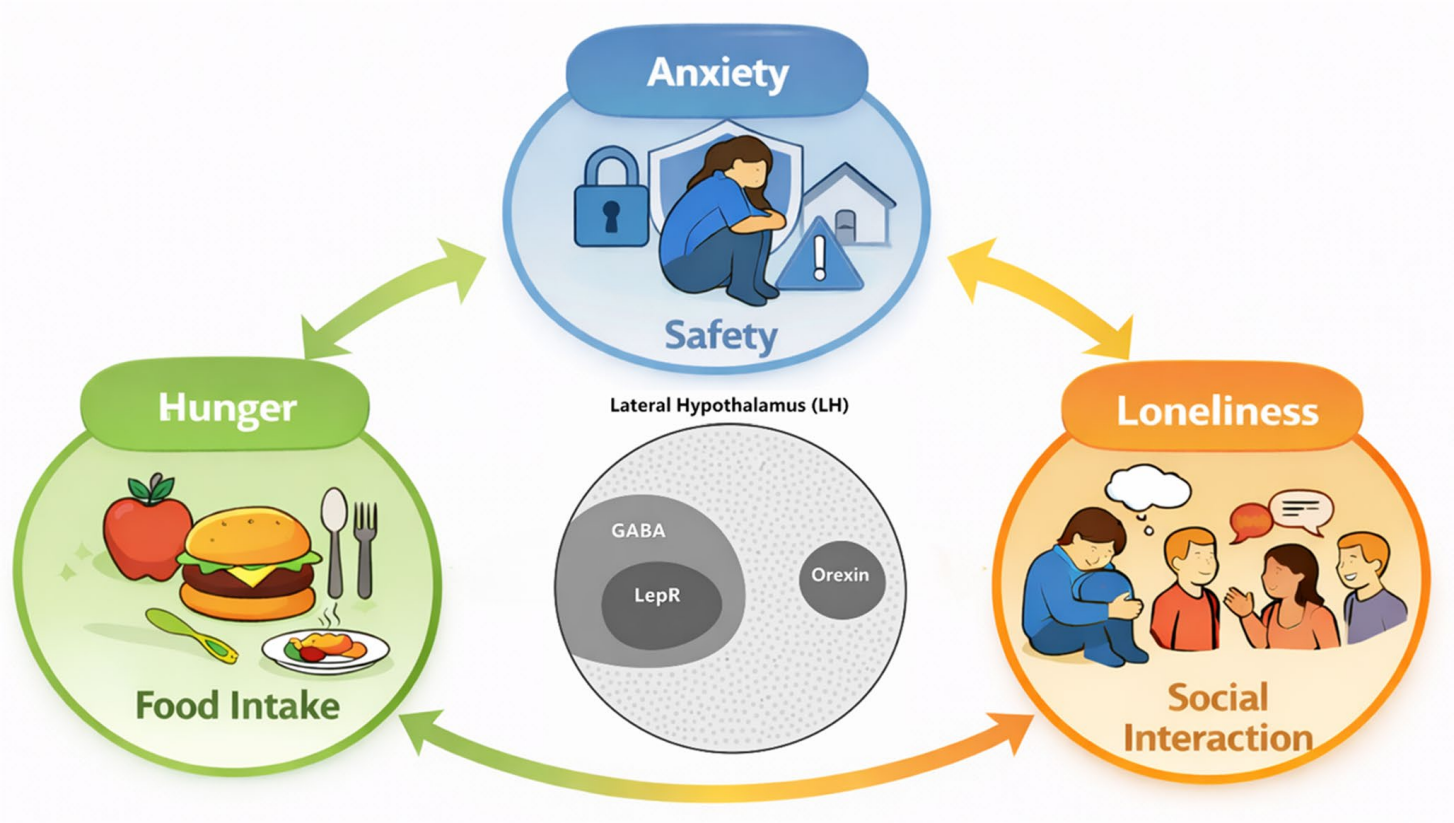
Competing motivational states underlying food intake, anxiety, and social behavior

Historically, the LH was conceptualized as a feeding center, based on classic lesion [1] or electrical stimulation [2] studies showing profound effects on food intake. This view has since expanded dramatically. The LH is now recognized as a highly heterogeneous structure composed of intermingled neuronal populations [3, 4] defined by neurochemical identity, connectivity, firing dynamics, and functional roles. Beyond feeding, LH circuits influence arousal, reward seeking, stress responses, locomotion, and social behavior [5–11]. This review focuses primarily on the functions of GABAergic and leptin receptor-expressing (LepR) neurons in the LH, based largely on evidence from circuit studies in rodents. Early electrical stimulation experiments already hinted at this broader role: stimulation of the LH could elicit a wide range of innate behaviors [5], reflecting the engagement of partially overlapping cell populations involved in feeding, sleep, social interaction, exploration, and appetitive motivation [5, 12, 13]. Classical electrophysiological studies showed that LH neurons respond to multiple innate rewards, aversive and conditioned stimuli. Because of its robust capacity to induce self-stimulation, LH was designated a „pleasure center“ [14], although it was reported that prolonged or strong stimulation may induce aversion [15]. This apparent paradox fits with the broader principle that aversion and reward represent complementary aspects of motivational control. Aversive states drive behaviors that ultimately lead to reward and fulfill crucial for survival needs: anxiety promotes actions that enhance safety, hunger motivates food seeking to restore energy balance, and loneliness encourages social interaction and mating (Fig. 1). LH activity can regulate motivational value, where the same circuitry can promote approach under some conditions yet drive avoidance under others, depending on neuronal activity, inputs, and the internal state of the organism. Further cell-type specific studies demonstrated multiple functions of the same cell type. For example, activation of LH GABA cells elicits feeding [12, 16, 17], awakening [18], hunting [19] and compulsory behaviors [13, 20]. These findings implied that overlapping LH populations participate in multiple behavioral programs, challenging the notion of discrete, behavior-specific modules. Instead, the LH appeared capable of dynamically allocating behavioral resources across domains.

## The lateral hypothalamus mediates behavioral transitions

Brain control mechanism for switching to a new instinctive behaviour was proposed in seminal ethological studies [21]. Recent findings demonstrate that LH neuronal activity encodes a preparatory transition state preceding behavioral change. This state is characterized by beta-frequency (15–30 Hz) oscillations and the recruitment of “transition cells” that are active across multiple behaviors. These neurons transiently co-activate with behavior-preferring populations, forming oscillation phase-dependent representations of alternative upcoming behaviors, such as feeding, social interaction and novel object exploration [22]. Disruption of this dynamic abolishes behavioral transitions, locking animals into ongoing actions such as continuous feeding or persistent social pursuit [22]. These results identify the LH as a key node for selecting and initiating behavioral switches rather than merely executing individual behaviors. Notably, this preparatory transition state, supported by beta oscillations, emerges in the LH approximately two seconds prior to behavior onset [22], indicating an early stage of commitment to behavioral change. This hypothalamic preparatory activity complements cortical preparatory signals described in classical human studies, in which activity in the supplementary motor cortex occurs less than one second before specific self-initiated actions (~ 0.8 s) [23] and even before conscious awareness of the action (~ 0.2 s before the movement onset) [24]. In addition to oscillatory dynamics at beta frequencies, faster synchronization in the LH also contributes to behavioral control. Gamma oscillations (30–90 Hz) are selectively enhanced during food approach and are required for effective food-seeking behavior [17]. Consistent with this, the LH regulates multiple stages of feeding, including food approach [17] and the learning of food-associated cues [25, 26]. Further, the LH orchestrates switching between hunting, which is necessary for feeding in many species, and evasion: activation of LH GABAergic projections to the periaqueductal gray (PAG), a midbrain structure instrumental to fear expression ([27] reviewed in [28]) promotes predatory behavior, whereas activation of LH glutamatergic projections to the same brain region drives evasion [19]. This hypothalamic coordination of switching between hunting and escape has also been demonstrated in humans [29]. Effects of LH GABA cells’ stimulation on feeding and reward are frequency specific [20]. This might be due to frequency-specific release of neuropeptides [5], described also for other neuronal populations [30, 31] or to frequency-specific coordination between brain regions [17, 22]. LH is strongly and bilaterally connected with the ventral tegmental area (VTA), a brain region critically implicated in reward processing ([16, 20], reviewed in [5]). The LH–VTA circuit makes projection-specific contributions to reward-related behaviors, with LH→VTA neurons promoting learned reward seeking independently of reward availability, and GABAergic LH inputs to the VTA strongly driving feeding-related behavior [16] in a frequency-dependent way [20]. These observations indicate that distinct LH output pathways subserve different components of motivational control, with LH projections to the PAG mediating predatory versus evasive responses, and LH projections to the VTA supporting reward-related, feeding-associated, and behavioral activation processes. Taken together, these results identify the LH as a key node for selecting and initiating behavioral switches rather than merely executing individual behaviors.

A defining feature of adaptive behavior is the capacity to resist the pressure of one need in order to fulfill another. LH circuits play a central role in this process by dynamically adjusting the priority of competing drives.

## Resisting hunger to pursue social opportunities

Hunger powerfully motivates feeding, yet animals must sometimes suppress feeding to pursue social or reproductive goals. A subset of LH GABA neurons expresses receptors for leptin, a hormone secreted by adipose tissue [32]. Leptin receptor-expressing LH (LepR) neurons provide a mechanism for such flexibility. While constitutive disruption of LepR signaling leads to hyperphagia and obesity in humans and animal models [32], and knockout of Lepr in LH increases food intake [33, 34], acute manipulations of LepR LH neurons demonstrate state-dependent functions of these neurons. While activation of LepR LH neurons has little effect on food intake in sated animals in a familiar environment [9, 35–37], it decreases food intake in mice with a moderate hunger but not with strong hunger elcitied by a prolonged food deprivation [9]. Further, leptin-activated LH neurons increase their activity during food approach specifically following acute, but not following prolonged food restriction or ad libitum access to food. This subpopulation may prevent the onset of feeding and could provide a potential substrate to limit over-feeding after acute fasting [9]. What is the function of this specific food intake supression during moderate hunger? Intraperitoneal leptin injections increase social interaction [9], enhance social interaction-induced place preference in non-stressed adolescence mice and reversed social anhedonia, elicited by chronic unpredictable stress [38] as well as promoted mating behaviors [39] in rodents. We found that LepR neurons promote social interaction with female mice despite food or water deprivation, suggesting that LepR neurons in the LH may provide a neural substrate for leptin’s pro-social effects. Further, these neurons encode conspecifics of the opposite sex, showing increased activity during interactions with males in female mice and with females in male mice [9]. Future studies will be required to identify the projection targets and co-expression profile of LepR LH neurons (e.g. co-expression with estrogen receptors was shown for a subpopulation of LepR cells for hypothalamic nuclei [40]), that mediate these pro-social and pro-mating functions of this cell population. Further LH cell population regulating social behavior are orexin/hypocretin-expressing neurons, which have a crucial role in arousal and vigilance [41]. They are activated in response to social interaction, especially with a novel mouse [42]. Their projections to the anterior cingulate cortex (ACC) are recruited during both affective empathy and prosocial behavior and control theta oscillations in ACC [43]. At the same time, activation of orexin LH neurons projections to the lateral habenula promotes male-male aggression and conditioned place preference for aggression-paired contexts [44]. These findings suggest a state-dependent regulation of social behaviors by orexin neurons. Further, LH neurons projecting to lateral habenula are activated by an unexpected social status loss but not a natural loss, suggesting state-dependent coding of social rank changes by this pathway [45].

## Overcoming anxiety to meet essential needs

Social and reproductive behaviors are strongly influenced by anxiety and perceived threat, which can suppress social interaction and mating. Previous studies in rodents suggested possible function of leptin as antidepressant [46, 47], and injections of leptin in VTA reduced anxiety-related behaviors in mice [48]. Leptin-deficient ob/ob mice show increases in anxiety-related behaviors [49] and have elevated corticosterone levels that can be reduced by leptin replacement [50]. Leptin levels are associated with decreased depressive symptoms in women across the weight spectrum, independent of body fat [51]. Anxiety serves the fundamental need for safety, analogous to hunger signaling metabolic need. However, anxiety can also hinder the fulfilling of crucial needs such as feeding or mating. To balance these competing demands, animals and humans need to overcome anxiety and meet essential needs despite anxiogenic conditions. We found that LepR LH neurons were activated by anxiogenic stimuli, such as the open arms of an elevated plus maze as well as by food presented under an anxiogenic condition. Opto- or chemogenetic activation of LepR LH neurons reduces anxiety-related behaviors [52]. Whereas baseline LepR LH activity is similar between animals with high- and low- levels of anxiety, the stimulus-evoked LepR LH responses differ between these groups of mice. In animals with low anxiety, LepR LH neurons reliably distinguish between anxiogenic stimuli, whereas in animals with high anxiety this discriminative capacity is reduced, potentially impairing stimulus-specific behavioral responses and promoting maladaptive behaviors. Further, an increase in LepR LH activity during food approach predicts successful feeding under anxiogenic conditions in animals with high anxiety, and experimental activation of LepR LH neurons selectively facilitates feeding in anxiogenic contexts [52]. These mechanisms are critical for survival in natural environments, where food acquisition often occurs under threat of predation. Increasing starvation pressure shifts prey species toward higher-risk foraging strategies [53, 54], reflecting flexible reprioritization of safety and feeding. LepR neurons inhibit orexin (hypocretin) neurons in the LH [55], and stress-induced activation of orexin neurons is reduced by leptin treatment [56], suggesting antagonistic interactions between leptin and orexin in the LH in regulation of stress responses and anxiety-related behaviors. Orexin (hypocretin) neurons, located exclusively in the LH, integrate metabolic signals with stress and arousal systems to coordinate adaptive behavioral and physiological responses. These neurons sense circulating metabolites and hormones, allowing them to link energy status to arousal, feeding, and motivated behaviors [57, 58]. In parallel, orexin signaling interacts with neuroendocrine systems regulating stress and metabolic homeostasis, including activation of the hypothalamic-pituitary-adrenal (HPA) axis and modulation of multiple endocrine pathways [59]. Further, orexins play an important regulatory role of the growth hormone functions by inhibiting its secretion modulating GHRH and somatostatin (SST) neurons [60, 61]. Beyond behavioral regulation, orexin signaling contributes to energy expenditure through central control of thermogenesis. Orexin modulates brown adipose tissue thermogenesis [62], thereby promoting thermogenesis and metabolic activation [63]. The thermogenic effect of bone morphogenetic protein 8B (BMP8B), a protein which increases brown adipose tissue thermogenesis through both central and peripheral actions [64], is mediated by the increase in orexin signaling, and the thermogenic effect of BMP8B is absent in orexin-null mice [65], highlighting a functional interaction between these systems in the regulation of energy expenditure. Through these mechanisms, orexin neurons link metabolic status to state-dependent arousal. Disruption of orexin signaling destabilizes wakefulness and produces narcolepsy type 1, a disorder caused by loss of orexin-producing neurons that leads to excessive daytime sleepiness and abnormal REM-sleep transitions [66, 67]. Further, activation of orexin neurons is necessary for developing a panic-prone state in the rat panic model, and human subjects with panic anxiety have elevated levels of orexin in the cerebrospinal fluid [68]. Glutamatergic projections from LH to locomotor-promoting pedunculopontine nucleus promote safety-seeking behavior over other essential needs such as foraging or social contact [69].

LepR subpopulations in the LH exhibit considerable heterogeneity, both in their co-expression of additional molecular markers and in their behavioral functions. For example, distinct LepR LH subpopulations regulate different stages of feeding behavior, including food approach and the consumption [9, 70, 71]. One subpopulation co-expresses Tac1+, the precursor of substance P, which has also been implicated in the regulation of anxiety. Mutations affecting Tac1 [72] or its receptor [73], as well as pharmacological blockade of Tac1 signaling [73], reduce anxiety- and depression-related behaviors, whereas treatment with Tac1 receptor agonists increases anxiety [73]. Another LepR population co-expresses galanin [74], a neuropeptide implicated in the regulation of anxiety. Consistent with this role, genetic variation in the GAL locus has been linked to the severity of anxiety disorders in women [75], and treatment with galanin, or chemogenetic activation of galanin LH neurons, reduces anxiety-like behavior in rodents [76–78]. A subset of LepR LH neurons also co-expresses neurotensin and corticotropin-releasing hormone (Crh) [79], yet activation of neurotensin LH neurons does not alter anxiety-related behaviors [52], suggesting that the anxiolytic effects are specific to LepR LH neurons that do not co-express neurotensin.

## When balance fails: implications for disease

Failures of balance between anxiety and feeding are evident in several neuropsychiatric and metabolic disorders. A pathological bias toward safety- and control-related drives at the expense of feeding is often observed in individuals with anorexia nervosa. They often show heightened anxiety, exaggerated threat sensitivity, and strong cognitive control over feeding behavior [80], suggesting a dysregulated balance between circuits encoding energy needs and those promoting avoidance or vigilance. Eating disorders show high comorbidity with anxiety disorders [81, 82], and enhanced vulnerability to stress, and anorexia nervosa onset is frequently preceded or exacerbated by stressful life events [83, 84]. Anorexia nervosa is marked by high relapse rates, with stress commonly acting as a precipitating factor [85]. Anxiety can drive maladaptive behaviors, including hyperactivity, as observed in anorexia nervosa, where a substantial fraction of patients engage in excessive exercise to alleviate negative affective states [86]. Rodent models further support the idea that anxiety-related processes can affect feeding: in the activity-based anorexia (ABA) model, food restriction combined with unlimited access to a running wheel produces excessive hyperactivity and suppressed feeding despite severe energy deficit. Importantly, anxiety levels correlate with the degree of hyperactivity in this model [87, 88], suggesting that maladaptive prioritization of safety-related circuits may drive excessive locomotor activity and continued food restriction. Consistent with this, exercise had anxiolytic effects in the ABA model [89]. Further, mice carrying genetic variant that elevate trait anxiety displayed exaggerated stress-induced hyperactivity [90].

Leptin treatment reduced hyperactivity and depressive symptoms in anorexia nervosa patients [91], and suppressed excessive exercise in the activity-based anorexia rodent model (ABA) [92] suggesting that leptin signaling can counteract these maladaptive responses and highlighting how disrupted metabolic signaling can contribute to pathological behavioral allocation. LepR LH neurons co-express anorexia risk genes *Opcml* (Opioid-binding protein/cell adhesion molecule) [93], as well as *Ebf1* (Early B-Cell Factor 1), mutations of which are also associated with anxiety disorders [93, 94]. Chemogenetic activation of LepR LH neurons abolishes excessive exercise in ABA model [52]. Importantly, LepR LH neurons regulate locomotion in the state-dependent, adaptive manner. LepR LH activation does not change locomotion in baseline, prior to exposure of animals to food restriction in anorexia nervosa model [52], or in an open field [95], but increases locomotion in sated animals in a novel enclosure [36] and in hungry animals searching for food [37]. These findings suggest that LepR LH neurons integrate metabolic and emotional state to flexibly gate locomotor output, promoting context-appropriate exploration or restraint. Identification of the projection-defined circuits mediating these actions will be an important goal for future studies.

Whereas excessive prioritization of safety and control may contribute to anorexia nervosa [80], obesity and binge-eating disorders may reflect a pathological shift toward excessive prioritization of feeding and reward-related signals. Stress, a potent driver of anxiety, profoundly alters feeding behavior, promoting binge eating in some individuals and hypophagia in others. Chronic stress exposure can remodel neural responses to food-associated cues, biasing behavior toward high-calorie food seeking and increasing vulnerability to obesogenic eating patterns [96]. Such stress-driven remodeling may alter the balance between hypothalamic circuits encoding metabolic need and those processing environmental reward cues, thereby promoting compulsive or cue-driven feeding even in the absence of energy deficit. Disruptions in the ability to flexibly prioritize and transition between behaviors are characteristic of many neuropsychiatric disorders. Further, eating disorders frequently co-occur with social avoidance [81], while autism spectrum disorders are often accompanied by altered eating behaviors [97]. These comorbidities suggest shared underlying circuit dysfunctions affecting behavioral arbitration rather than isolated deficits in single motivational systems. Because the LH integrates metabolic, emotional, and social information to arbitrate between competing behavioral drives, disruptions in LH circuit function may represent a common substrate underlying these diverse clinical conditions. Understanding how LH populations dynamically coordinate feeding, anxiety, and social behaviors may therefore provide important insight into the circuit mechanisms linking metabolic disorders with broader psychiatric pathology.

## Beyond neurochemical identity: multidimensional coding in the lateral hypothalamus

**Fig. 2:**
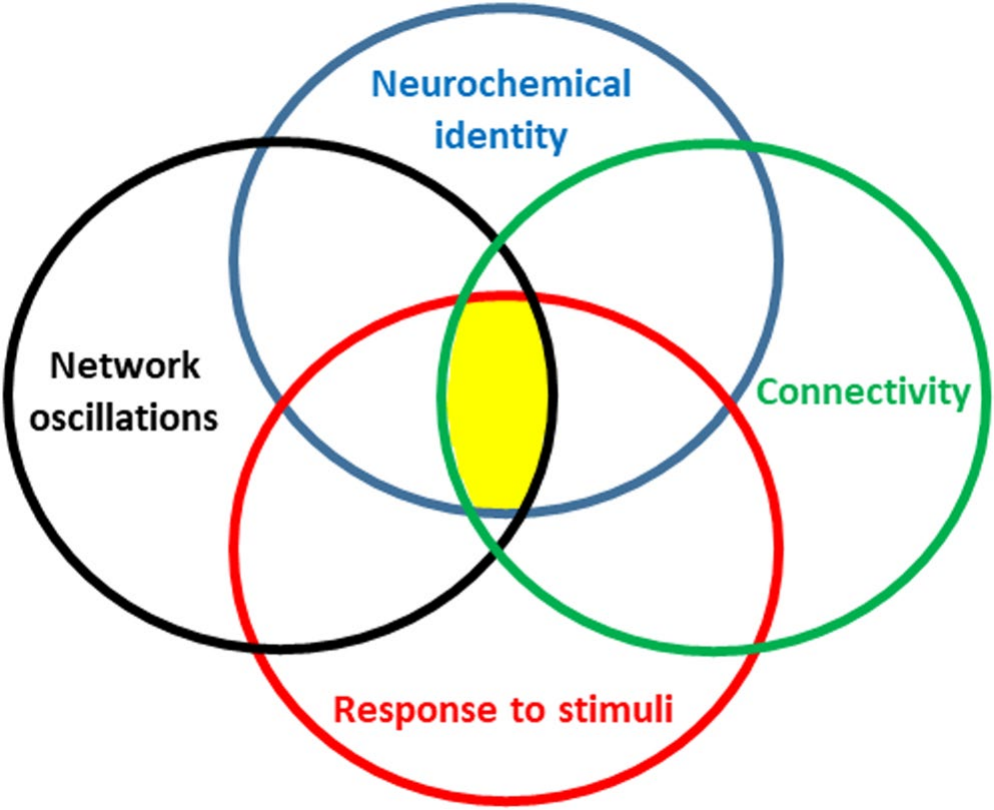
Combination of features defines unique identity of LH neuorns

This review is mainly focused on functions of several LH populations (Box 1). Yet LH neuronal identity emerges from a combination of features, including neurochemical markers, oscillatory phase, synaptic inputs, and responses to stimuli (Fig. 2). Cells with similar neurochemical identities can demonstrate different connectivity [3, 4, 98], be active at different phases of network oscillations [17, 22], or display distinct responses to stimuli [5]. This multidimensional coding adds a new layer to hypothalamic organization and enables the same population to participate in different behaviors depending on context. We propose that behavioral output is determined by the convergence of these features rather than by single molecular labels. Such an organization provides the flexibility required to coordinate complex behaviors and rapidly adapt to changing demands.

 Box 1. The lateral hypothalamus contains heterogeneous neuronal populations defined by molecular identity and behavioral functions

**Table:** 

LH neuronal population	Behavioral functions	Notes
GABAergic LH neurons	Feeding, reward seeking, behavioral activation, predation	Activation can elicit feeding, hunting, locomotion, and compulsive behaviors; effects are frequency-dependent
Glutamatergic LH neurons	Evasion, defensive responses, safety-seeking	Functionally oppose LH GABA projections in predation-evasion switching and regulation of feeding
LepR-expressing LH neurons (subset of GABA neurons)	Suppression of feeding during moderate hunger, promotion of social interaction, reduction of anxiety	Integrate metabolic state with emotional and social signals; enable animals to overcome anxiety to pursue feeding or social behavior
Orexin/hypocretin neurons	Arousal, vigilance, social interaction, aggression, metabolic regulation	Integrate metabolic and stress signals; regulate BAT thermogenesis and endocrine responses; loss of orexin neurons causes narcolepsy

## Conclusions and outlook

The LH is emerging as a central coordinator of multiple innate behaviors and transitions between them. By dynamically balancing competing needs for food, safety, and social interaction, LH circuits enable animals to resist the pressure of one drive in order to fulfill another. This flexibility is essential for survival, yet vulnerable to disruption in disease. Dynamic signaling in the LH upon changing metabolic and environmental demands achieves balanced output and generates, maintains and adaptively changes consistent adaptive behavior. Understanding how LH populations integrate internal state, environmental context, and social information to orchestrate behavior will be critical for uncovering the neural basis of adaptive behavior. Several important questions remain regarding how individual LH neurons integrate multiple streams of information to guide behavioral prioritization. In particular, it will be critical to understand how metabolic, emotional, and social signals converge at the level of single cells and how projection-specific circuits dynamically coordinate transitions between competing behaviors. Advances in single-cell recording, projection-specific manipulation, and transcriptomic profiling will help clarify how multidimensional features, such as molecular identity, connectivity, and oscillatory phase, combine to shape LH function. A better understanding of these mechanisms may also reveal new therapeutic opportunities, as LH circuits represent potential intervention points for disorders characterized by maladaptive prioritization of feeding, anxiety, or social behaviors, including eating disorders, anxiety disorders, and stress-related conditions. Given the prominent involvement of these processes in eating disorders, anxiety disorders, and social dysfunction, LH circuits represent promising targets for future therapeutic strategies.

## Data Availability

No datasets were generated or analysed during the current study.
